# Immune response to the severe acute respiratory syndrome coronavirus 2 vaccines: Is it sustained in the diabetes population?

**DOI:** 10.1111/jdi.13787

**Published:** 2022-03-25

**Authors:** Erasmia Rouka, Eleni Livanou, Sotirios Sinis, Ilias Dimeas, Ioannis Pantazopoulos, Dimitrios Papagiannis, Foteini Malli, Ourania Kotsiou, Konstantinos I Gourgoulianis

**Affiliations:** ^1^ 37786 Department of Respiratory Medicine Faculty of Medicine University of Thessaly Larissa Greece; ^2^ 37786 Faculty of Nursing University of Thessaly Larissa Greece

## DISCLOSURE

The authors declare no conflict of interest.

Approval of the research protocol: Approval was obtained from the Research Ethics Committee of the University Hospital of Larissa, Greece. The procedures used in this study adhere to the tenets of the Declaration of Helsinki.

Informed consent: Informed consent was obtained from all study participants.

Approval date of registry and registration no. of the study/trial: N/A.

Animal studies: N/A.

Previous studies have highlighted the interrelation between coronavirus disease 2019 (COVID‐19) and diabetes: COVID‐19 has a deleterious effect on the diabetes population, and diabetes can induce severe COVID‐19 outcomes^1^, ^2^. Here, we report the substudy results of our COVAX study involving patients hospitalized at the COVID‐19 Department of the University Hospital of Larissa, Greece, who were fully vaccinated with a severe acute respiratory syndrome coronavirus 2 (SARS‐CoV‐2) messenger ribonucleic acid or an adenoviral vector deoxyribonucleic acid vaccine, to assess the possible impact of diabetes on the maintenance of vaccine‐induced SARS‐CoV‐2 immunoglobulin G antibodies.

A total of 92 patients with laboratory‐confirmed SARS‐CoV‐2 infection were enrolled in the substudy (62 men, 30 women). The institutional review board approved the study (46943/29.11.2021), and written informed consent was obtained from each participant involved. Serum samples were collected from each patient on the first day of hospitalization for the semiquantitative determination of the SARS‐CoV‐2 immunoglobulin G spike (S) protein‐specific antibodies with lateral flow immunochromatographic assays having 96.72% and 100% diagnostic sensitivity and specificity, respectively (Catalog number: V1430; ProGnosis Biotech S.A., Larissa, Greece). Participants were classified in two groups: cases with a negative antibody test and cases with detectable antibody levels.

**Figure 1:**
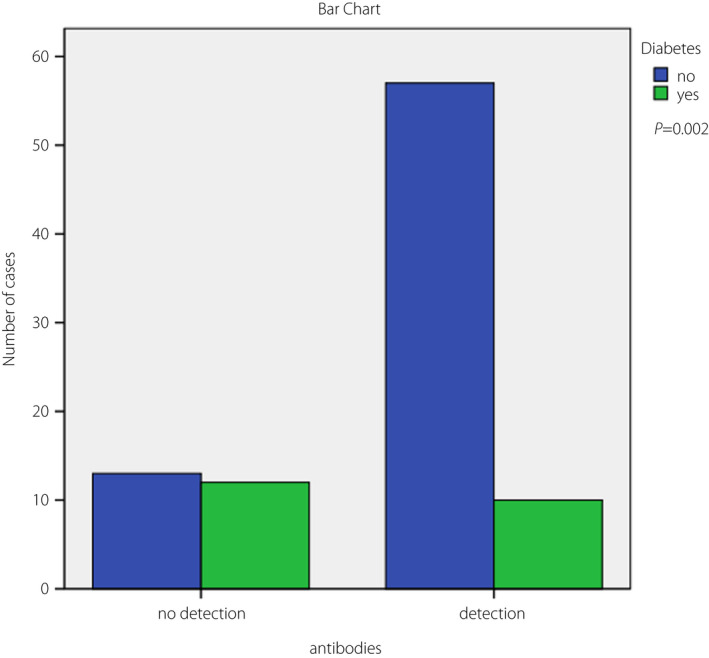
Frequency of diabetes in fully vaccinated hospitalized coronavirus disease 2019 patients, classified in cases with a negative or positive SARS‐CoV‐2 Spike immunoglobulin G antibody test on admission. A total of 25 patients did not have detectable antibodies, of whom 12 had diabetes. Out of the 67 patients that had detectable antibodies, just 10 had diabetes.

A total of 25 participants had a negative antibody test on admission. The absence of anti‐S SARS CoV‐2 antibodies was associated with a shorter time from symptom onset to hospitalization (4.04 ± 0.5 days vs 6.51 ± 0.3 days, *P *< 0.001). A total of 22 participants had a confirmed diagnosis of diabetes and were receiving treatment. The mean age of participants was 71.73 ± 1.5 years in the non‐diabetes group and 73.65 ± 2.1 years in the diabetes group (*P *= not significant). The mean number of days since completion of vaccination in the two aforementioned subgroups was 154.40 ± 7.8 and 173.76 ± 9.5, respectively (*P* = not significant). The frequency of diabetes was significantly higher in patients who did not have detectable antibodies on admission (*P *= 0.002; Figure 1).

The present study was limited to COVID‐19 hospitalized patients. However, the findings raise concerns regarding the maintenance of SARS‐CoV‐2 vaccine immunity in adults with diabetes. Two recent studies have reported conflicting results regarding the association between anti‐SARS‐CoV‐2 antibody levels and glycemic control^3^, ^4^. Further research is warranted to estimate the longevity of immune responses after SARS‐CoV‐2 vaccination in patients with diabetes.
